# Trends in Methadone Use for Pain and Opioid Use Disorder Among Medicaid Enrollees

**DOI:** 10.1001/jamahealthforum.2025.5023

**Published:** 2025-11-21

**Authors:** Yu-Chia (Sam) Hsu, Ashish P. Thakrar, Charles E. Leonard, Margaret Lowenstein, Colleen M. Brensinger, Warren B. Bilker, Kacie F. Bogar, Sean Hennessy

**Affiliations:** 1 Center for Real-World Effectiveness and Safety of Therapeutics, Department of Biostatistics, Epidemiology, and Informatics, University of Pennsylvania Perelman School of Medicine, Philadelphia; 2 Division of General Internal Medicine, Department of Medicine, University of Pennsylvania Perelman School of Medicine, Philadelphia; 3 Center for Addiction Medicine and Policy, University of Pennsylvania Perelman School of Medicine, Philadelphia; 4 Leonard Davis Institute of Health Economics, University of Pennsylvania, Philadelphia

## Abstract

This cross-sectional study aims to quantify methadone use for opioid use disorder in the Medicaid program and assesses buprenorphine use as a possible shift between medications for opioid use disorder.

## Introduction

In recent decades, policies have aimed to increase access to methadone and buprenorphine for opioid use disorder (OUD), as these medications have the strongest evidence for reducing mortality and improving OUD treatment retention.^1^ Use of medications for OUD (MOUD) such as methadone and buprenorphine might therefore be expected to increase.

However, prior studies show mixed results.^2,3,4^ State-level analyses found slight decreases in methadone use for OUD in Medicaid,^2^ and a national study inferred a decline in methadone use for OUD among Medicaid enrollees based on methadone prescriptions following Medicaid expansion.^3^ Conversely, another study found that the amount of methadone, in kilograms, distributed to opioid treatment programs (OTPs) increased over the past decade, suggesting increased use for OUD.^4^ Notably, US regulations prohibit methadone prescribing for OUD outside OTPs; methadone for outpatient OUD treatment may only be administered or dispensed by OTPs.

To reconcile these divergent findings, this study aimed to quantify methadone use for OUD in the Medicaid program, the largest US insurer of persons with OUD, covering 38% of this population.^2^ Buprenorphine use was also included to assess possible shifts between MOUD.

## Methods

We measured the annual number of patients receiving methadone or buprenorphine for OUD among adult (≥18 years old) Medicaid enrollees from 1999 to 2020 using a 100% dataset from the Centers for Medicare & Medicaid Services, which includes all 50 states; Washington, DC; Puerto Rico; and the Virgin Islands. Individuals dually enrolled in Medicare and Medicaid were excluded to reduce data incompleteness.

Methadone treatment for OUD via OTPs was identified via Healthcare Common Procedure Coding System codes, while methadone prescribed for pain was identified by National Drug Codes (NDCs; eMethods in Supplement 1). This distinction was possible because of US regulations restricting methadone for OUD treatment to OTP settings and allowing methadone prescriptions only for the treatment of pain. For buprenorphine products, we restricted the analysis to US Food and Drug Administration–approved formulations for OUD. The size of the annual US Medicaid adult population was obtained from the Medicaid and Children’s Health Insurance Program Payment and Access Commission data book.^5^

University of Pennsylvania’s institutional review board approved the study with a waiver of informed consent. We followed the Strengthening the Reporting of Observational Studies in Epidemiology (STROBE) reporting guidelines.

## Results

There were modest increases in methadone and buprenorphine use per 1000 Medicaid enrollees from 1999 to 2010 (Figure). After 2010, the prevalence of buprenorphine use for OUD rose substantially to 12.0 per 1000 Medicaid enrollees, while methadone use for OUD grew a lesser degree to 6.2 per 1000 Medicaid enrollees. In contrast, methadone prescribed for pain declined after 2010 to 0.4 per 1000 Medicaid enrollees.

**Figure.**
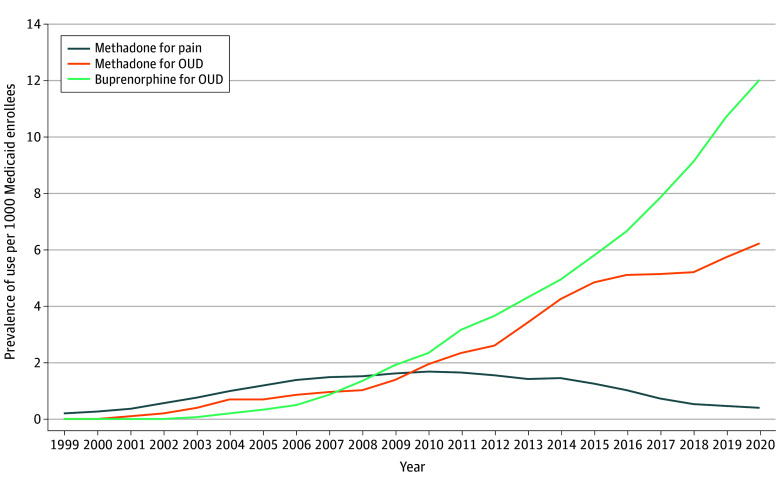
Trends in Methadone and Buprenorphine Use by Indication Among Medicaid Enrollees, 1999-2020 By 2020, the prevalence of buprenorphine use for opioid use disorder (OUD) was 12.0 users per 1000 Medicaid enrollees, methadone use for OUD was 6.2 users per 1000 Medicaid enrollees, and methadone use for pain was 0.4 users per 1000 Medicaid enrollees. Persons may be counted more than once if they were exposed to more than 1 of these medications in the same year.

## Discussion

This repeated cross-sectional study demonstrates that methadone and buprenorphine use for OUD continues to grow among Medicaid enrollees. Although buprenorphine use was nearly twice that of methadone in 2020, methadone use for OUD did not decline as previous studies suggested.^2,3^ State variation in methadone coverage and limited inclusion of states (ie, representing 22% of Medicaid enrollees^2^ vs a 100% sample in the present study) may explain differing methadone use trends for OUD. A prior study that found declining methadone use used NDC prescription codes,^3^ which likely reflects methadone use for pain rather than for OUD. This aligned with results of this study when identifying methadone prescription claims via NDC codes. Further studies on methadone use for OUD should use Healthcare Common Procedure Coding System codes. This study was limited by possible incompleteness of Medicaid data from the Centers for Medicare & Medicaid Services.

Despite rising MOUD use, unmet need remains, as only 25% of individuals with OUD receive MOUD.^6^ Given recent policy efforts—such as the Substance Abuse and Mental Health Services Administration’s expansion of methadone take-home doses and the proposed Modernizing Opioid Treatment Access Act—further research on the effects of expanding authorized methadone prescribers and permitting methadone prescriptions via pharmacies will be critical to improving MOUD access.

## Notes

Supplement 1.
eMethods

Supplement 2.
Data Sharing Statement
