# Supplementary material for: Trends in Methadone Use for Pain and Opioid Use Disorder Among Medicaid Enrollees
Source: JAMA Health Forum. 2025 Nov 21;6(11):e255023. doi: 10.1001/jamahealthforum.2025.5023 (PMC12639476; doi:10.1001/jamahealthforum.2025.5023)
Supplement: Supplement 1. — eMethods [file jamahealthforum-e255023-s001.pdf]

Supplemental Online Content

Hsu YC, Thakrar AP, Leonard CE, et al. Trends in methadone use for pain and opioid use disorder among Medicaid enrollees. *JAMA Health Forum*. 2025;6(11):e255023. doi:10.1001/jamahealthforum.2025.5023

eMethods

This supplemental material has been provided by the authors to give readers additional information about their work.

© 2025 Hsu YC et al. *JAMA Health Forum*.

eMethods

1. HCPCS codes to identify methadone administration for OUD treatment

H0020: Alcohol and/or drug services; methadone administration and/or service

S0109: Methadone, oral, 5 mg

G2078: Take-home supply of methadone; up to 7 additional day supply

G2067: Medication assisted treatment, methadone; weekly bundle for OTPs

2. HCPCS codes to identify buprenorphine administration for OUD treatment

J0570: Buprenorphine implant, 74.2 mg

J0571: Buprenorphine, oral, 1 mg

J0572: Buprenorphine/naloxone, oral, less than or equal to 3 mg buprenorphine

J0573: Buprenorphine/naloxone, oral, greater than 3 mg, but less than or equal to 6 mg buprenorphine

J0574: Buprenorphine/naloxone, oral, greater than 6 mg, but less than or equal to 10 mg buprenorphine

J0575: Buprenorphine/naloxone, oral, greater than 10 mg buprenorphine

G2068: Medication assisted treatment, buprenorphine (oral); weekly bundle for OTPs

G2069: Medication assisted treatment, buprenorphine (injectable); weekly bundle for OTPs

G2079: Take-home supply of buprenorphine (oral) for OTPs; list separately in addition to code for primary procedure.

Q9991: Injection, buprenorphine extended-release, less than or equal to 100 mg

Q9992: Injection, buprenorphine extended-release, greater than 100 mg

3. NDC codes to identify FDA approved buprenorphine products for OUD

Buccal film of buprenorphine/naloxone

59385001201, 59385001230, 59385001401, 59385001430, 59385001601, 59385001630

Sublingual tablet/film of buprenorphine/naloxone:

00054018813, 00054018913, 00093572056, 00093572156, 00121101830, 00121203630, 00228315403, 00228315409, 00228315473, 00228315503, 00228315509, 00228315573, 00378876593, 00378876693, 00378876793, 00378876893, 00406800503, 00406802003, 00490005100, 00490005130, 00490005160, 00490005190, 00781721664, 00781722764, 00781723864, 00781724964, 00904700906, 00904701006, 12496010001, 12496030001, 12496120203, 12496120403, 12496120803, 12496121203, 12496128302, 12496130602, 16729054910, 16729055010, 35356000430, 42291017430, 42291017530, 42858060103, 42858060203, 43063018407, 43063018430, 43598057930, 43598058030, 43598058130, 43598058230, 47781035503, 47781035603, 47781035703, 47781035803, 49999039515, 50268014415, 50268014515, 50383028793, 50383029493, 50742036430, 50742036530, 51862060830, 52959030430, 52959074930, 54123011430, 54123090730, 54123091430, 54123092930, 54123095730, 54123098630, 54569573900, 54569573901, 54569573902, 54868570700, 54868575000, 55887031204, 55887031215, 55887031230, 55887031290, 60429058630, 60429058633, 60429058730, 60429058733, 60687062665, 60687063765, 62175045232, 62175045832, 62756096983, 62756097083, 63629948201, 63874108403, 63874108503, 65162041503, 65162041603

Sublingual tablet/film of buprenorphine mono products :

© 2025 Hsu YC et al. *JAMA Health Forum*.

00054017613, 00054017713, 00093537856, 00093537956, 00121101930, 00121203830, 00228315303, 00228315603, 00378092393, 00378092493, 00904715404, 00904715504, 12496127802, 12496131002, 42858050103, 42858050203, 43063066706, 43063075306, 49999063830, 49999063930, 50268012815, 50268012915, 50383092493, 50383093093, 60687048121, 60687049221, 62756045983, 62756046083, 63629947501, 63874117303, 68308020230, 68308020830, 71335115403, 72162134403, 72162134503, 72888018230, 72888018330

Extended-release injection of buprenorphine received FDA approval for opioid use disorder:

12496010001, 12496030001

4. NDC codes to identify methadone products for pain indication

00002106402, 00002107202, 00002168201, 00002168225, 00002215302, 00054070920, 00054070925, 00054071020, 00054071025, 00054121811, 00054121842, 00054355367, 00054355467, 00054355663, 00054421625, 00054421725, 00054454725, 00054854725, 00406052705, 00406151056, 00406151057, 00406151059, 00406345434, 00406412303, 00406412310, 00406622505, 00406697434, 00527192736, 00904653060, 00904741761, 13107008801, 13107008901, 17478038020, 23490587703, 23490587706, 23490587707, 23490587709, 23490587801, 23490587802, 23490587803, 23490587809, 23490779801, 23490779803, 31722094601, 31722094701, 38779010403, 42806031701, 42806031801, 43063022260, 43063022290, 43063022293, 43063022298, 49999083930, 49999083960, 49999083990, 49999084030, 49999084060, 49999084130, 49999084160, 49999096330, 49999096360, 49999096390, 51079069439, 52959043502, 52959043560, 52959043590, 54868285400, 54868440800, 54868494800, 54868494801, 54868494802, 54868494803, 54868494804, 54868494805, 54868494806, 54868570100, 55289081430, 55289081460, 55289081490, 55289081493, 55289081498, 55289081499, 55887009030, 55887009060, 55887009090, 55887013460, 55887013490, 55887020001, 55887020005, 55887020010, 55887020020, 55887020030, 55887020042, 55887020050, 55887020056, 55887020060, 55887020090, 60687020932, 60687021401, 60687081886, 61553012078, 61553013490, 61553014978, 61553016063, 63629102701, 63629209801, 63629378801, 63739000610, 63874126503, 63874126506, 64019053825, 64019055367, 64019055467, 64019075085, 64019075088, 66336017062, 66336017090, 66336017094, 66336017160, 66336017162, 66336017190, 66336017194, 66336017198, 66479053002, 66591081551, 66689068155, 66689069430, 66689069479, 66689069579, 66689071116, 66689071216, 66689081010, 66689082010, 66689083699, 66689089840, 67457021720, 67877011601, 68084073801, 68084097732, 68094003162, 68094003258, 68462080001, 68462080101, 70004038005, 70092116143, 70092157343, 72865012001, 72865012101, 73177014943, 73177016143, 00054039168, 00054039268, 00054355344, 00054355563, 00054421825, 00054421925, 00054453825, 00054457025, 00054457125, 00054855324, 00054855424, 00406052710, 00406054034, 00406254001, 00406575501, 00406575562, 00406577101, 00406577162, 00406872510, 00904653061

===

Note. When calculating the annual prevalence of methadone or buprenorphine use in Medicaid (i.e., the number of patients with methadone or buprenorphine claims per adult Medicaid enrollees), except for methadone prescribed for pain, patients (i.e., the numerator) were required to have an OUD diagnosis at any time prior to receiving methadone or buprenorphine for OUD (using International Classification of Diseases, Ninth Revision [ICD-9] codes 304.0x, 305.5x, 304.7x, and ICD-10 codes F11.x).

© 2025 Hsu YC et al. *JAMA Health Forum*.
