# Supplementary material for: Trends in Methadone Use for Pain and Opioid Use Disorder Among Medicaid Enrollees
Source: JAMA Health Forum. 2025 Nov 21;6(11):e255023. doi: 10.1001/jamahealthforum.2025.5023 (PMC12639476; doi:10.1001/jamahealthforum.2025.5023)
Supplement: Supplement 2. — Data Sharing Statement [file jamahealthforum-e255023-s002.pdf]

# Data Sharing Statement

Hsu. Trends in Methadone Use for Pain and Opioid Use Disorder Among Medicaid Enrollees. *JAMA Health Forum*. Published November 21, 2025. doi:10.1001/jamahealthforum.2025.5023

**Data**
**Data available:** No

**Additional Information**
**Explanation for why data not available:** If these findings arose from our use of CMS data on the VDRC, we are held to a restrictive data use agreement and are not permitted by HHS to share. Interested persons can contact ResDAC, a CMS contractor.
